# 1261. Prospective Audit and Feedback Approach Differs between Antimicrobial Stewardship Professions

**DOI:** 10.1093/ofid/ofad500.1101

**Published:** 2023-11-27

**Authors:** Sara Ausman, Kristin Mara, Caitlin Brown, Kevin L Epps, Kirstin Kooda, Julio C Mendez, Christina G Rivera (O'Connor)

**Affiliations:** Mayo Clinic Health System - Eau Claire, Eau Claire, Wisconsin; Mayo Clinic, Rochester, Minnesota; Mayo Clinic, Rochester, Minnesota; Mayo Clinic Florida, Jacksonville, Florida; Mayo Clinic - Rochester, Rochester, Minnesota; Mayo Clinic Florida, Jacksonville, Florida; Mayo Clinic, Rochester, Minnesota

## Abstract

**Background:**

Implementing effective antimicrobial stewardship interventions is an important component of antimicrobial stewardship programs. Recently, stewardship research has shown that clinician characteristics may have an impact on antimicrobial stewardship intervention methods and acceptance rates.

**Methods:**

A multisite health system antimicrobial stewardship prospective audit and feedback program interventions performed by stewardship staff using a standardized electronic medical record (EMR) tool were compared by stewardship clinician profession (pharmacist, physician, or advanced practice provider, APP) from 7/1/2017 to 6/30/2022. Outcomes assessed were intervention rates, communication methods, and intervention acceptance by patient age and intensive care unit (ICU) status of patient. Generalized estimating equations utilizing logistic regression were used to assess for associations between staff. Associations were assessed both univariately and after adjusting for other factors.

**Results:**

Of 69,503 prospective audit and feedback EMR based rules, there were 14,699 rules associated with an intervention. The majority of rules were reviewed by pharmacists (90.7%) followed by physician (9.2%) and APP (< 1%). Of 9,034 interventions with an outcome documented, 7326 (85.5%) pharmacist interventions were accepted and 360 (77.3%) physician interventions were accepted (adjusted p=0.047).  Pharmacists intervened by direct action (ie, adjust a medication order) in 6.6% of interventions; whereas, physicians did so rarely (0.4%). Pharmacists were more likely to use synchronous communication than physicians in the adjusted model (pharmacist vs. physician: 63.8% vs 52.5%; p=0.004). Patients in the ICU had a significantly lower intervention acceptance rate (non-ICU vs. ICU: 86.6% vs. 78.0%; adjusted OR 0.57 [95% CI 0.46-0.70], p< 0.001).

**Table 1.**
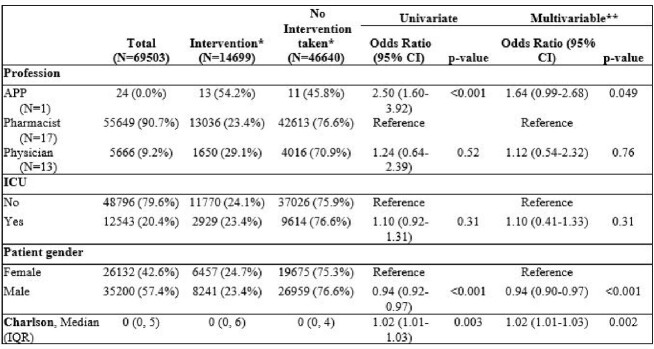
Intervention Rate by Baseline Demographics of Stewardship Clinicians and Patients.

*% are reported as row % **Multivariable model was also adjusted for day of week and time of day APP-Advanced practice provider; ICU-intensive care unit

**Table 2.**
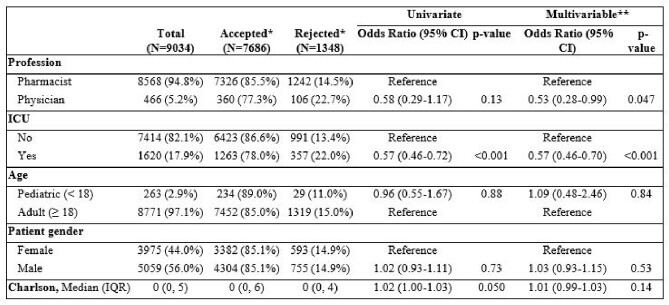
Intervention Acceptance.

*% are reported as row % **Multivariable model was also adjusted for day of week and time of day APP-Advanced practice provider; ICU-intensive care unit

**Conclusion:**

Antimicrobial stewardship pharmacists and physicians are both effective at prospective audit and feedback but used different intervention styles. Advanced practice providers were vastly underrepresented. Patients in the ICU may benefit from tailored ASP efforts.

**Disclosures:**

**Sara Ausman, PharmD**, Gilead: Honoraria **Caitlin Brown, PharmD**, Alexion: Grant/Research Support|Trevena: Advisor/Consultant **Christina G. Rivera (O'Connor), Pharm.D**, Gilead Sciences: Advisor/Consultant|Gilead Sciences: Board Member|Gilead Sciences: Grant/Research Support|Gilead Sciences: Honoraria

